# Stronger hearts, weaker leaps? The cardiac power paradox in elite soccer

**DOI:** 10.1007/s00421-025-06026-3

**Published:** 2025-10-18

**Authors:** Zacharias Papadakis, Nikolaos Koutlianos, Vassilios Panoutsakopoulos, Evangelia Kouidi

**Affiliations:** 1 Human Performance Laboratory, Department of Health Sciences and Clinical Practice, College of Health Professions and Medical Sciences, Barry University, Miami Shores, FL 33161 USA; 2 Laboratory of Sports Medicine, School of Physical Education and Sports Science at Thessaloniki, Aristotle University of Thessaloniki, Thessaloniki, Greece; 3 Biomechanics Laboratory, School of Physical Education and Sports Science at Thessaloniki, Aristotle University of Thessaloniki, 54124 Thessaloniki, Greece

**Keywords:** Athlete’s heart, Left ventricular hypertrophy, Anaerobic power, Repetitive vertical jump test, Cardiac remodeling, Soccer performance, Neuromuscular adaptation

## Abstract

**Abstract:**

While elite soccer cultivates concurrent cardiovascular and neuromuscular adaptations, the functional interplay between exercise-induced left ventricular hypertrophy (LVH) and explosive power remains underexplored.

**Purpose:**

The purpose of the study was to determine if left ventricular mass index (LVMI), a primary marker of cardiac adaptation, is associated with explosive power assessed via a repetitive vertical jump test (RVJT).

**Methods:**

Nineteen elite male soccer players underwent 2D echocardiography for LVMI and a 15-repetition RVJT on a force plate for maximum jump height (h_MAX_). The relationship was tested using hierarchical regression (controlling for body mass and experience), Bayesian analysis, and group comparisons based on the clinical LVH threshold (≥ 115 g/m^2^).

**Results:**

An inverse LVMI–h_MAX_ relationship was observed (*r* =  − 0.53, *p* = 0.02). In the adjusted model, LVMI showed *B* =  − 0.00123 m per g·m⁻^2^ (SE 0.00046; 95% CI [− 0.00220 to − 0.00026]; standardized *β* =  − 0.63; *p* = 0.02); after Benjamini–Hochberg correction across five RVJT outcomes, *q* = 0.10. Athletes with high LVMI exhibited 17% lower h_MAX_ than their normal-LVMI counterparts (0.29 ± 0.04 vs. 0.35 ± 0.04 m; *p* = 0.003, Hedges’ *g* =  − 1.51), with moderate Bayesian support (*BF*_10_ = 3.47). Other RVJT parameters were unaffected.

**Conclusion:**

The findings are preliminary and hypothesis generating, consistent with a potential trade-off between cardiac remodeling and maximal explosive performance in elite male soccer. Greater cardiac mass is associated with attenuated explosive power capacity, a functional “cardio-neuromuscular paradox.” The RVJT may serve as a practical tool to monitor this systemic balance and inform training adjustments to preserve power in athletes with pronounced cardiac remodeling.

**Supplementary Information:**

The online version contains supplementary material available at 10.1007/s00421-025-06026-3.

## Introduction

Chronic exercise induces profound cardiac remodeling, with left ventricular hypertrophy (LVH) representing a key adaptation to volume-load stress in endurance sports (Pelliccia et al. 2025). However, in disciplines requiring concurrent aerobic and anaerobic capacity, like elite professional soccer, the functional consequences of LVH on explosive power remain unknown. Modern soccer is characterized by a dualistic stimulus: prolonged aerobic efforts, with players routinely covering > 10 km per match, are punctuated by frequent explosive bursts, such as 5–30 m sprints and other rapid decelerations, over 90 min (Boraczynski et al. 2020; Nobari et al. 2023). This stimulus forces profound adaptations across players’ cardiovascular and neuromuscular systems (Boraczynski et al. 2020; Hostrup and Bangsbo 2023; Morrison et al. 2023). Across a season, intensified training further amplifies these demands, provoking measurable changes in both physiological domains (Francavilla et al. 2018; Green et al. 2024; Nobari et al. 2023). While these pathways are well documented in isolation, their functional interplay remains insufficiently characterized.

On the cardiovascular front, chronic exposure to soccer’s unique activity profile imposes what is effectively a hybrid hemodynamic load on the heart (Baba Ali et al. 2024; Petek et al. 2022). High-volume, sustained running produces a persistent volume overload that promotes eccentric hypertrophy to enhance diastolic filling and stroke volume, whereas frequent, intermittent high-intensity sprints, jumps, and changes of direction create transient pressure overload that stimulates concentric hypertrophy to manage acute increases in wall stress (Churchill et al. 2021; Morrison et al. 2023; Pelliccia et al. 2025; Unnithan et al. 2024; von Lueder et al. 2018). The integration of these stimuli remodels the myocardium, leading to the well-documented “athlete’s heart,” often presenting as an increase in left ventricular mass (LVM) (Churchill et al. 2021; Johnson et al. 2023; Starekova et al. 2020).

The combined mixed-training elements in soccer induce a physiological left ventricular hypertrophy (LVH) that is structurally and functionally distinct from pathological forms (Barauna et al. 2007; Kurtoglu et al. 2024; Pittaras et al. 2023). To quantify physiological hypertrophy and distinguish it from pathology, contemporary methods scale LVM to body size, typically using a left ventricular mass index (LVMI) threshold of approximately 115 g/m^2^ (Oxborough et al. 2024). This remodeling appears cumulative and dose-dependent; a ~ 6% rise in LVMI was observed after just 15 weeks of recreational soccer in adults (Sjuretharson et al. 2024), while 18 months of elite academy training increased LVMI by ~ 8% in youth players (Unnithan et al. 2024). Although largely deemed physiological, this hypertrophy may have performance-relevant implications: each additional gram of myocardium raises total body mass and the energetic cost of movement, and higher LVMI can coexist with attenuated myocardial strain, suggesting a structural-to-functional trade-off (Johnson et al. 2023; Oxborough et al. 2024; Starekova et al. 2020). These factors may impede rapid neuromuscular responses essential to explosive match-play tasks (Green et al. 2024; Morrison et al. 2023).

Concurrently, from a neuromuscular perspective, a soccer match demands dozens of vertical accelerations, aerial duels, changes of direction, and rapid decelerations that rely on the efficiency of the stretch–shortening cycle (SSC) (Suarez-Arrones et al. 2020). This explosive capacity can be reliably captured by the repetitive maximal vertical jump test (RVJT) (Theodorou et al. 2013). Methodological work confirms that RVJT metrics, maximum jump height (h_MAX_), average height (h_AVE_), reactive strength index at the highest jump (RSI_MAX_), and relative power (P_REL_), derived from force plates, exhibit near-perfect reliability and explain meaningful variance in sprinting and change-of-direction performance, which supports their use as readiness markers in elite settings (Bishop et al. 2023, 2022; Montalvo et al. 2021; Stratford et al. 2020). Furthermore, these jump-derived indices can discriminate between academy players who secure professional contracts (Papadakis et al. 2022a, b, c) and are correlated with other anaerobic proxies like Wingate peak power (Boraczynski et al. 2020).

Conceptually, these cardiovascular and neuromuscular adaptations have traditionally been viewed as separate, parallel phenomena, under a siloed perspective (Di Gioia et al. 2025; Pelliccia et al. 2025; Squeo et al. 2025; Zholshybek et al. 2023). Treating them as independent may overlook a system-level interaction consistent with the established interference effect of concurrent training, where simultaneous endurance and strength stimuli can attenuate explosive performance gains (Arslan et al. 2025; Huiberts et al. 2024; Hung et al. 2025; Jurasz et al. 2022; Petre et al. 2021; Schumann et al. 2022; Seipp et al. 2022; Vechin et al. 2021). We use trade-off to denote a plausible mechanistic exchange between endurance-dominant and power-dominant adaptations, and paradox to denote the counterintuitive organism-level observation that a marker of favorable cardiovascular adaptation may coexist with lower maximal explosive performance. At a hypothetical mechanistic level, endurance-type stimuli that promote cardiac remodeling activate AMP-activated protein kinase signaling (AMPK), whereas explosive power development relies more on mammalian target of rapamycin mTOR-dependent protein synthesis; mutual inhibition between these pathways could create a systemic milieu less conducive to maximal power development (Egan and Sharples 2023; Furrer and Handschin 2024). Because these pathways can be mutually inhibitory, the systemic physiological environment that fosters long-term cardiac remodeling may be less conducive to the neuromuscular adaptations required for maximal power development. This potential for a functional trade-off, which we term a “cardio-neuromuscular performance paradox,” can be conceptualized as a specific, organism-level manifestation of the broader interference effect, since an enlarged ventricle may augment stroke volume (Green et al. 2024; Morrison et al. 2023; Sjuretharson et al. 2024) and RVJT performance correlates with established anaerobic proxies (Boraczynski et al. 2020; Nobari et al. 2023).

This prompts the key question of whether a systemic manifestation of the concurrent training interference effect is observable at the whole organism level. If profound structural remodeling of the heart is governed by signaling pathways that can interfere with neuromuscular adaptation, it is plausible that this cardiac phenotype is functionally coupled to neuromuscular performance. To date, no peer-reviewed study has explicitly investigated whether this chronic cardiac adaptation is associated with, predicts variance in, or is neutral to RVJT-derived anaerobic power in elite soccer players, despite competing cardiovascular and neuromuscular demands (Di Gioia et al. 2025; Pelliccia et al. 2025; Squeo et al. 2025). To address this gap, the present study examined whether LVMI, a key marker of chronic cardiac adaptation, independently explains variance in repetitive vertical jump performance in elite male soccer players (Bishop et al. 2023; Oxborough et al. 2024). Grounded in the preceding evidence, we hypothesized that higher LVMI, reflecting a more pronounced endurance-type cardiac adaptation, would be inversely associated with key metrics of explosive performance, consistent with a potential physiological trade-off between central (cardiac) and peripheral (neuromuscular) performance.

## Methods

### Participants

A cross-sectional observational study design with criterion sampling was employed to investigate the relationship between LVMI, derived from echocardiography measurements, and indices of explosive anaerobic performance derived from the RVJT. All players completed the same anthropometric, echocardiographic, and 15-repetition repetitive vertical jump test (RVJT) protocol under standardized conditions.

To test the hypothesis of the study, 19 adult Greek male Super League professional soccer players were examined at the beginning of the pre-season period. Inclusion criteria were the participation in international top-level competitions and their involvement in systematic training. Exclusion criteria were severe injury and/or health problems that prevented training or competition for six months prior to testing. All participants provided signed informed consent. The study was conducted in accordance with the Declaration of Helsinki and the Institution’s Research Committee Ethics Code, and approval was granted by the Research Committee of Aristotle University of Thessaloniki, Greece (#281/07.24.25).

### Experimental procedures

#### Echocardiographic measurements

After basic anthropometrics—specifically, body mass, body height, and body surface area (BSA), a clinical evaluation including medical history and resting 12-lead electrocardiogram was performed for all participants. The Vivid S70 (GE Medical; Horten, Norway) with an M5 S phased-array transducer was used to perform echocardiographic studies for each athlete by an experienced cardiologist-ultrasonographer. Two-dimensional (2D) echocardiography was used to evaluate the athlete's heart. A comprehensive assessment of right and left ventricular systolic and diastolic structure and function was conducted. Left ventricular ejection fraction (LVEF) was calculated using the modified biplane Simpson method, while left ventricular mass (LVM) was assessed using the truncated ellipsoid technique and indexed to BSA (LVMI) (Ahmadi et al. 2023; Roberto M Lang et al. 2015a, b; Nagueh et al. 2016; Olsen et al. 2022; Oxborough et al. 2024) Images were stored in EchoPAC, version 204 and subsequently analyzed in a random order, to avoid bias, by two experienced cardiologists in compliance with the European Association of Cardiovascular Imaging and American Society of Echocardiography guidelines (Arbelo et al. 2023; Heidenreich et al. 2022; Nagueh et al. 2016). Formal inter- and intra-observer reproducibility statistics (e.g., ICC) were not computed in this dataset and are acknowledged as a methodological limitation.

#### Biomechanical measurements

Following the echocardiographic measurements, all participants executed 15 continuous maximal effort countermovement jumps on a prototype 1.00 × 1.00 m^2^ K-Force force plate (KINVENT, Biomecanique, Montpellier, France) to extract the RVJT performance parameters. The examined parameters were the maximum (h_MAX_) and average (h_AVE_) jump height, the average (RSI_AVE_) and reactive strength index at the highest jump (RSI_MAX_), and the relative power output (P_REL_) were extracted according to current best-practice guidelines (Bishop et al. 2022). The procedure for the data acquisition and analysis for the anthropometric variables and the RVJT performance has been described in detail in a previous study (Papadakis et al. 2022a, b, c). Briefly, during the RVJT, vertical ground-reaction forces (V_GRF_) were captured at a sampling frequency of 1 kHz. The raw force data were processed with a low-pass filter to minimize measurement error. From the V_GRF_ time series, ground contact time (t_C_) and flight time (t_FL_) were determined for each jump. These data were then used to calculate key performance outcomes: jump height (h_JUMP_) was estimated from t_FL_, and reactive strength index (RSI) was calculated as the ratio of h_JUMP_ to t_C_ (Panoutsakopoulos et al. 2022). The average relative power output (P_REL_) was determined according to the Bosco et al. formula (Bosco et al. 1983). Mean values for h_JUMP_, RSI and P_REL_ across all 15 jumps were used for the final analysis.

### Statistical analysis

Unless stated otherwise, tests were two-tailed with *α* = 0.05. The primary analytical approach treated LVMI as a continuous predictor variable to maximize statistical power and preserve the full variance in the data. This avoids the loss of information that occurs when continuous variables are dichotomized, a critical consideration in studies with smaller sample sizes where statistical power is paramount (Abt et al. 2020; Gupta et al. 2022; Morera et al. 2023). To account for potential confounders, a set of anthropometric, body composition, and training history parameters were recorded. Body mass and training experience were selected a priori as covariates: body mass for its direct mechanical influence on jump performance, and training experience as a proxy for cumulative exposure that may influence cardiac remodeling. Lean mass was considered but showed high collinearity with total mass in this sample (*r* > 0.90), so total mass was retained to avoid multicollinearity (Ishida et al. 2021; Smaranda et al. 2025; Thompson et al. 2017; Uzomba et al. 2025; Yang et al. 2023; Zimmermann et al. 2022). For clinical context, a secondary analysis was also conducted by dichotomizing participants into two groups using the established threshold for left ventricular hypertrophy (LVH): a high-LVMI group (LVMI ≥ 115 g/m^2^) and a normal-LVMI group (LVMI < 115 g/m^2^) (R. M. Lang et al. 2015a, b; Most et al. 2024; Oxborough et al. 2024).

#### Data preparation

All analyses were conducted using JASP (version 0.19.3, Apple Silicon) (Han and Dawson 2020) which was selected for its robust statistical capability including the user-friendly Bayesian analysis option (Berkhout et al. 2024; Coon et al. 2025; Kangiwa et al. 2024; Walker et al. 2021). Normality of RVJT outcomes (e.g., h_MAX_, h_AVE_, RSI_AVE_, RSI_MAX_, P_REL_) and LVMI was assessed using Shapiro–Wilk tests and Q–Q plots. Non-normal variables were analyzed with non-parametric tests (e.g., Mann–Whitney *U*, Spearman’s correlations). Homogeneity of variances was tested using Levene’s test for group comparisons. Potential outliers were flagged with box- and raincloud plots and the 1.5 × IQR criterion and retained unless indicative of measurement error. Missing data, if < 5%, were handled via listwise deletion; if ≥ 5%, expectation–maximization imputation was applied in JASP. Descriptive statistics (mean ± SD for continuous variables, *n* [%] for categorical variables) were computed for participant characteristics (e.g., age, height, body mass, BMI, training experience, body fat%, body surface area, lean body mass) and RVJT outcomes, stratified by the LVMI group (Group A: LVMI ≥ 115 g/m2; Group B: LVMI < 115 g/m2). Between-group differences in baseline characteristics were tested using independent *t*-tests (or Mann–Whitney *U* for non-normal data) for continuous variables and Chi-square tests for categorical variables to ensure group comparability (Norskov et al. 2021).

#### Sample size considerations and power

A post hoc power analysis was performed with G*Power 3.1.9.6 (test family: F tests; statistical test: Linear multiple regression, fixed model, R2 increase)(Faul et al. 2009, 2007). With a medium incremental effect size (f2 = 0.15), α = 0.05, one tested predictor (LVMI) in a three-predictor model (LVMI, body mass, training experience) and N = 19, the achieved power was 0.35. An a priori calculation for the same parameters indicated that 55 participants would be required to reach 80% power. This cross-sectional study, which employed purposive sampling to recruit elite athletes, was underpowered for detecting medium or smaller incremental effects. Recognizing the challenges in accessing this specialized population, which limited sample size and representativeness, we prioritized the interpretation of results through effect sizes (standardized *β*, Hedges’ *g*, Spearman’s *ρ*) and their 95% confidence intervals (Cohen 2013, 2016; Murphy et al. 2025a, b).

#### Primary analysis: hierarchical multiple regression

To address the research question, whether LVMI independently explains variance in functional anaerobic power, hierarchical multiple regression was performed for each RVJT outcome (h_AVE_, h_MAX_, RSI_AVE_, RSI_MAX_, P_REL_), reflecting its integration of force and velocity. LVMI (continuous, g/m2) was the primary predictor, with body mass (kg) and training experience (years) as covariates to control for confounding effects on neuromuscular performance (Van Dusen and Nissen 2019; Wang et al. 2025).

- *Step 1* Body mass and training experience were entered to account for baseline variance.
- *Step 2* LVMI was added to assess its unique contribution.

Standardized beta coefficients (β), change in R2 (ΔR2), and 95% confidence intervals (CIs) were reported to quantify the strength and direction of LVMI’s effect. Model diagnostics included variance inflation factors (VIF < 5) for multicollinearity, Durbin–Watson tests for residual independence, and residual plots for linearity and homoscedasticity. If normality assumptions were violated, bias-corrected and accelerated (BCa) bootstrap CIs (5000 resamples) were computed. To control for multiple testing across five RVJT outcomes, the Benjamini–Hochberg false discovery rate (FDR, *q* = 0.05) was applied (Murray and Blume 2021).

#### Secondary analysis: group comparison

To provide a clinically intuitive perspective, RVJT outcomes were compared between LVMI groups (Group A: LVMI ≥ 115 g/m^2^; Group B: LVMI < 115 g/m^2^) using independent *t*-tests for normally distributed outcomes or Mann–Whitney *U* tests for non-normal outcomes. Body mass and training experience were included as covariates in ANCOVA models if assumptions were met; otherwise, non-parametric comparisons were prioritized (Akritas 2025; Lüdtke and Robitzsch 2025; Mondal and Kumar 2024). Benjamini–Hochberg FDR (*q* = 0.05) was applied across the five RVJT outcomes. Effect sizes were reported as Hedges’ *g* (for *t*-tests with small-sample correction) or rank-biserial correlation (for Mann–Whitney *U*), with 95% CIs to quantify group differences (Cohen 2013, 2016).

#### Complementary analysis: correlational and Bayesian analysis

To explore the LVMI-RVJT relationship, Pearson’s *r*, Spearman’s rank-order correlations (*ρ*), or Kendall’s tau (*τ*) were computed (depending on the normality of the data) between LVMI and each RVJT outcome, given the small sample and potential non-normality. Partial correlations, controlling for body mass and experience, were also calculated (Fayad et al. 2025; Wisniewski and Brannan 2025). Bayesian, Pearson’s *r*, and Kendall’s *τ* correlation analysis were performed in JASP to quantify evidence for the hypothesized inverse relationship. Bayes factors (BF_10_) and 95% credible intervals were reported, with BF_10_ > 3 indicating moderate evidence for the alternative hypothesis and BF_10_ < 0.33 supporting the null (Huth et al. 2023).

## Results

### Participant characteristics

The study included 19 adult male professional soccer players. Participants were divided into two groups based on left ventricular mass index (LVMI): high-LVMI (n = 11, LVMI ≥ 115 g/m^2^) and normal-LVMI (n = 8, LVMI < 115 g/m^2^). Descriptive statistics for participant characteristics and RVJT outcomes are presented in Table 1. No significant differences were found in baseline characteristics (age, height, body mass, BMI, training experience) between groups (all p > 0.05).

**Table 1:** Descriptive statistics of participant characteristics and rvjt outcomes stratified by the LVMI group

Variable	Total (*n* = 19)	High LVMI (*n* = 11)	Normal LVMI (*n* = 8)	*P* value
Age (years)	25.66 ± 4.55	25.45 ± 4.11	25.95 ± 5.38	0.82
Height (m)	1.81 ± 0.07	1.82 ± 0.07	1.79 ± 0.08	0.40
Body mass (kg)	75.85 ± 7.27	76.70 ± 8.91	74.69 ± 4.46	0.57
Training experience (years)	11.53 ± 3.39	11.18 ± 3.46	12.0 ± 3.46	0.62
Body fat (%)	11.72 ± 2.18	11.33 ± 2.60	12.26 ± 1.41	0.37
BMI (kg/m^2^)	23.50 ± 0.48	23.41 ± 0.32	23.63 ± 0.65	0.35
BSA (m^2^)	1.96 ± 0.13	1.98 ± 0.14	1.94 ± 0.10	0.45
LBM (kg)	67.18 ± 6.90	68.29 ± 8.39	65.65 ± 4.16	0.62
LVMI (g/m^2^)	128.44 ± 23.05	143.14 ± 18.06	108.22 ± 9.66	0.0001
h_AVE_ (m)	0.25 ± 0.04	0.24 ± 0.03	0.27 ± 0.05	0.13
h_MAX_ (m)	0.31 ± 0.05	0.29 ± 0.05	0.35 ± 0.04	0.003
RSI_AVE_	1.49 ± 0.20	1.50 ± 0.17	1.48 ± 0.26	0.88
RSI_MAX_	1.86 ± 0.28	1.87 ± 0.29	1.86 ± 0.29	0.98
P_REL_ (W/kg)	10.78 ± 0.91	10.55 ± 0.71	11.10 ± 1.11	0.21

All values are presented as mean ± standard deviation

*P* values are from independent *t*-tests or Mann–Whitney *U* tests for non-normal variables

*RVJT* repetitive vertical jump testing, *BMI* body mass index, *BSA* body surface area, *LBM* lean body mass, *LVMI* left ventricular mass index, *h*_*AVE*_ average jump height, *h*_*MAX*_ maximum jump height, *RSI*_*AVE*_ average reactive strength index, *RSI*_*MAX*_ maximum reactive strength index, *P*_*REL*_ relative power

### Assumption checks

Shapiro–Wilk tests indicated that all variables were approximately normally distributed (all *p* > 0.05). Within groups, LVMI in the normal-LVMI group deviated from normality (*p* = 0.002). Levene’s tests confirmed homogeneity of variances for group comparisons (all *p* > 0.05). Collinearity diagnostics showed no issues (VIF < 1.3), and Durbin-Watson statistics indicated minimal autocorrelation (values ≈ 1–2).

### Primary analysis: hierarchical multiple regression

Hierarchical multiple regression examined whether LVMI independently predicted variance in RVJT outcomes (h_AVE_, h_MAX_, RSI_AVE_, RSI_MAX_, P_REL_), with body mass and training experience in Step 1 and LVMI added in Step 2 (Table 2). For maximum jump height (h_MAX_), adding LVMI significantly improved the model (ΔR2 = 0.31, *p* = 0.02). The standardized coefficient for LVMI was *β* =  − 0.63 (*p* = 0.02), and the unstandardized coefficient was* B* =  − 0.00123 m per g·m⁻2 (95% CI [− 0.0022, − 0.0003]), indicating that each 1 g·m⁻2 increase in LVMI corresponded to an approximate 0.12 cm decrease in h_MAX_. After Benjamini–Hochberg correction across the five RVJT outcomes, this result did not survive FDR correction (*q* = 0.10). For the other RVJT parameters (h_AVE_, RSI_AVE_, RSI_MAX_, P_REL_), adding LVMI did not significantly improve the models (all *p* > 0.05 for ΔR2), with standardized coefficients ranging from − 0.37 to 0.11 (all *p* > 0.05). Model diagnostics confirmed no multicollinearity (VIF < 1.3) or autocorrelation concerns (Durbin–Watson ≈ 2).

**Table 2:** Hierarchical multiple regression results for RVJT outcomes

Outcome	Step	Predictors	R^2^	ΔR^2^	p (ΔR^2^)	Standardized β (LVMI)	Unstandardized B (LVMI)	p (LVMI)	95% CI (LVMI)	FDR q value
h_AVE_	1	Mass, experience	0.03	–	–	–	–	–	–	
	2	+ LVMI	0.14	0.11	0.19	-0.37	–	0.19	[− 0.0017, 0.0004]	0.42
h_MAX_	1	Mass, experience	0.04	–	–	–	–	–	–	
	2	+ LVMI	0.35	0.31	0.02*	− 0.63	− 0.00123	0.02*	[− 0.0022, − 0.0003]	0.1
RSI_AVE_	1	Mass, experience	0.05	–	–	–	–	–	–	
	2	+ LVMI	0.05	0.00	0.97	− 0.01	–	0.97	[− 0.0054, 0.0052]	0.97
RSI_MAX_	1	Mass, experience	0.13	–	–	–	–	–	–	
	2	+ LVMI	0.14	0.01	0.69	0.11	–	0.69	[− 0.0057, 0.0084]	0.86
P_REL_	1	Mass, experience	0.02	–	–	–	–	–	–	
	2	+ LVMI	0.11	0.08	0.25	− 0.32	–	0.25	[− 0.04, 0.01]	0.42

*RVJT* repetitive vertical jump testing, *LVMI* left ventricular mass index, *h*_*AVE*_ average jump height, *h*_*MAX*_ maximum jump height, *RSI*_*AVE*_ average reactive strength index, *RSI*_*MAX*_ maximum reactive strength index, *P*_*REL*_ relative power

*Significant at 0.05; FDR: Benjamini–Hochberg false discovery rate. Unstandardized coefficients B for LVMI are in meters per g·m⁻^2^. FDR q values reflect Benjamini–Hochberg adjustment across five RVJT outcomes

### Secondary analysis: group comparison

Independent samples *t*-tests and ANCOVA were used to compare RVJT outcomes between the high-LVMI and normal-LVMI groups, controlling for body mass and training experience in ANCOVA models. Results are presented in Table 3. For h_MAX_, a significant difference was observed (t(17) = − 3.39, *p* = 0.003, Hedges’ *g* = − 1.51, 95% CI [− 2.61, − 0.51]), with the normal-LVMI group showing higher maximum jump heights (M = 0.35 ± 0.04 m) compared to the high-LVMI group (M = 0.29 ± 0.04 m). ANCOVA confirmed this effect (F = 12.49, *p* = 0.003, η^*2*^*p* = 0.45, 95% CI [0.08, 0.69]). No significant differences were found for h_AVE_, RSI_AVE_, RSI_MAX_, or P_REL_ (all *p* > 0.05), with effect sizes indicating minimal to moderate differences (Hedges’ g ranging from − 0.70 to 0.07).

**Table 3:** Group comparison results for RVJT outcomes

Outcome	High LVMI (M ± SD)	Normal LVMI (M ± SD)	*t*	*p*	Hedges’ g	95% CI	ANCOVA F	ANCOVA p	η^2^p	ANCOVA FDR *q* value
h_AVE_	0.24 ± 0.03	0.27 ± 0.05	− 1.58	0.13	− 0.70	[− 1.67, 0.22]	2.77	0.12	0.16	0.30
h_MAX_	0.29 ± 0.04	0.35 ± 0.04	− 3.39	0.003*	− 1.51	[− 2.61, − 0.51]	12.49	0.003*	0.45	0.015*
RSI_AVE_	1.50 ± 0.17	1.48 ± 0.26	0.15	0.88	0.07	[− 0.84, 0.98]	0.04	0.85	0.002	0.97
RSI_MAX_	1.87 ± 0.29	1.86 ± 0.29	0.03	0.98	0.01	[− 0.90, 0.93]	0.001	0.97	0.0002	0.97
P_REL_	10.55 ± 0.71	11.10 ± 1.11	− 1.31	0.21	− 0.58	[− 1.53, 0.33]	1.93	0.18	0.11	0.30

*RVJT* repetitive vertical jump testing, *LVMI* left ventricular mass index, *h*_*AVE*_ average jump height, *h*_*MAX*_ maximum jump height, *RSI*_*AVE*_ average reactive strength index, *RSI*_*MAX*_ maximum reactive strength index, *P*_*REL*_ relative power

*Significant at 0.05; FDR: Benjamini–Hochberg false discovery rate. FDR *q* values reflect Benjamini–Hochberg adjustment across outcomes

### Complementary analysis: correlations and Bayesian analysis

To further explore the relationship between cardiac structure and anaerobic power, both frequentist and Bayesian correlations were computed. Pearson (linear relationship), Spearman (monotonic relationship), and Kendall (monotonic relationship) correlations were computed to explore the relationships between the LVMI and RVJT outcomes. LVMI was significantly negatively correlated with h_MAX_ (Pearson’s *r* = − 0.53, *p* = 0.02, 95% CI [− 0.79, − 0.10]; Spearman’s *ρ* = − 0.52, *p* = 0.02, 95% CI [− 0.79, − 0.09]; and Kendall’s tau-*b* = − 0.36, *p* = 0.03, 95% CI [− 0.65, − 0.06]), but not with h_AVE_, RSI_AVE_, RSI_MAX_, or P_REL_ (all *p* > 0.05). Partial correlations, controlling for body mass and training experience, suggested a significant negative relationship for h_MAX_ (*r* = − 0.57, *p* = 0.02; *ρ* = − 0.55, p = 0.02; *τ* = − 0.37, *p* = 0.04), and no other significant correlations were observed between LVMI and the other RVJT outcomes. The Bayesian analysis provides additional context. The Bayesian correlation analysis provided moderate evidence for a negative correlation between LVMI and h_MAX_ (BF_10_ = 3.47), indicating that the observed data are approximately 3.5 times more likely under a model that includes an association between these variables than under a null model of no association. In contrast, for all other RVJT outcomes, the Bayes factors were below 1.0 (BF_10_ = 0.29–0.54), providing anecdotal evidence in favor of the null hypothesis (i.e., supporting a true lack of relationship).

## Discussion

The current investigation examined whether LVMI, a marker of cardiac adaptation, is associated with functional anaerobic power in elite male soccer players (Oxborough et al. 2024; Pelliccia et al. 2025; Squeo et al. 2025). After Benjamini–Hochberg adjustment across the five RVJT outcomes, the primary regression finding (LVMI predicting h_MAX_) did not survive FDR correction (*q* = 0.10) (Murray and Blume 2021). Interpreting the totality of evidence with appropriate caution, we note a consistent pattern: the uncorrected regression *(p* = 0.02; *β* =  − 0.63), the group comparison that remained significant after FDR (ANCOVA FDR *q* = 0.015), and moderate Bayesian support (BF_10_ = 3.47). This triangulation provides preliminary, hypothesis-generating support for an inverse LVMI–h_MAX_ association consistent with a cardio-neuromuscular performance paradox (Gutierrez et al. 2025; Hammerton and Munafo 2021).

Results indicated a moderate inverse relationship between LVMI and h_MAX_ across multiple analytical approaches. (a) In hierarchical multiple regression, after accounting for body mass and training experience, adding LVMI explained an additional 31% of variance in h_MAX_ (ΔR2 = 0.31; *β* =  − 0.63; *p* = 0.02), indicating that a one SD increase in LVMI corresponded to a 0.63 SD decrease in h_MAX_ (Table 2). (b) Group comparisons (*p* = 0.003; Hedges’ *g* =  − 1.51) provided a clinically intuitive view: players exceeding the clinical LVH threshold (≥ 115 g·m^−2^) displayed meaningfully lower h_MAX_ than their normal-LVMI counterparts (Table 3). (c) Correlational analyses (*r* =  − 0.53; *p* = 0.02) and a Bayesian test (BF_10_ = 3.47) likewise supported an inverse association (Table 4). Importantly, no significant associations emerged for h_AVE_, RSI metrics, or P_REL_, suggesting specificity to maximal jump height. Collectively, these results partially support the study hypothesis: athletes with larger left ventricles tended to exhibit lower maximal jump height.

**Table 4:** Correlation coefficients between LVMI and RVJT outcomes

Outcome	Pearson’s r	*p*	Spearman’s ρ	*p*	Kendall’s τ	*p*	Partial r (LVMI)	*p*	Bayes BF_10_
h_AVE_	− 0.28	0.24	− 0.27	0.26	− 0.15	0.36	− 0.34	0.19	0.54
h_MAX_	− 0.53*	0.02	− 0.52*	0.02	− 0.36*	0.03	− 0.57*	0.02	3.47
RSI_AVE_	− 0.10	0.70	− 0.20	0.42	− 0.17	0.33	− 0.01	0.97	0.30
RSI_MAX_	− 0.03	0.91	− 0.13	0.59	− 0.09	0.63	0.10	0.69	0.29
P_REL_	− 0.23	0.34	− 0.22	0.37	− 0.13	0.44	− 0.29	0.25	0.43

*RVJT* repetitive vertical jump testing, *LVMI* left ventricular mass index, *h*_*AVE*_ average jump height, *h*_*MAX*_ maximum jump height, *RSI*_*AVE*_ average reactive strength index, *RSI*_*MAX*_ maximum reactive strength index, *P*_*REL*_ relative power

*Significant at 0.05

### The interference effect

We use interference effect as the overarching concurrent training construct, reserve paradox for the observed counterintuitive organism-level pattern, and employ trade-off to denote a plausible physiological exchange underlying the association. The enlarged “athlete’s heart,” particularly increased myocardial mass, contributes to overall body mass (Maxwell and Oxborough 2025). While echocardiographic indexing (i.e., LVMI) is designed to correct for body surface area, it does not negate the physical reality that a heavier heart is part of a heavier system that must be propelled against gravity during explosive movements like jumping. We speculate that each gram of hypertrophied, non-contractile (in the locomotor sense) cardiac tissue may impose an energetic penalty on the neuromuscular system, which must overcome this added mass. Increased cardiac mass adds ≈300 g to total body mass in our LVH subgroup; when repeated 15 times in rapid succession, the energetic overhead may reach a threshold where contractile power is marginally but detectably diluted. Furthermore, chronic volume overload could shift fiber-type composition toward a slower, oxidative phenotype, attenuating rapid force production (Green et al. 2024; Methenitis et al. 2025). In our data, the high-LVMI group, despite similar body mass to the normal-LVMI group (Table 1), exhibited markedly lower h_MAX_. These observations are compatible with a physiological trade-off between cardiac remodeling and explosive neuromuscular performance, as adding LVMI accounted for a sizable increment in h_MAX_ variance (ΔR2 = 0.31) (Di Gioia et al. 2025; Jurasz et al. 2022; Pelliccia et al. 2025; Saunders et al. 2022; Squeo et al. 2025; Stoickov et al. 2023).

The specificity to h_MAX_, but not other parameters (h_AVE_ or RSI metrics), may reflect h_MAX_ sensitivity to peak force production, maximal rate of force development and peak velocity of the SSC which could be hindered by physiological changes associated with higher LVMI, such as increased body mass, training intensity, or altered muscle fiber dynamics (Barrio et al. 2023; Cmentowski et al. 2023; Gradidge and Constantinou 2018; Liu et al. 2021; Methenitis et al. 2025; Weberruss et al. 2022). In contrast, h_AVE_ and RSI may better reflect fatigue resistance or elastic energy reuse across repeated efforts (Akyildiz et al. 2022; Lourenco et al. 2023; Munoz-Gracia et al. 2025; Ramirez-Campillo et al. 2023). Thus, a high-LVMI milieu might preferentially dampen the velocity component of the power–velocity profile, blunting the absolute peak of explosive capacity while leaving endurance-related qualities comparatively (Egan and Sharples 2023; Schumann et al. 2022; Solberg et al. 2025).

Soccer’s dualistic nature stimulus of concurrent and combined mixed-modality training presents an interference effect between the elicited adaptations of simultaneously aerobic and anaerobic training (Green et al. 2024). Aerobic exercise/training activates the AMP-activated protein kinase signaling pathway which governs the mitochondrial biogenesis and enhances oxidative capacity and altering cardiac morphology and function (Abrego-Guandique et al. 2025; Egan and Sharples 2023; H. Zhang et al. 2024a, b; X. Zhang et al. 2024a, b). Conversely, anaerobic exercise/training stimulates the mammalian target of rapamycin pathway that in return promotes the muscle protein synthesis and hypertrophy (Callahan et al. 2021; Lim et al. 2022; Roberts et al. 2023; Solsona et al. 2021; Thomas et al. 2024). Hypothetically, an endurance-dominant milieu that incrementally increases LVMI (via AMPK-related signaling and sustained volume/output) may be less conducive to mTOR-driven adaptations essential for maximizing SSC function and peak power output (Dziedzic et al. 2025; Furrer and Handschin 2024; Levitt et al. 2022; Steinberg and Hardie 2023).

### Context within existing literature

To our knowledge, this is the first study to directly quantify the association between an echocardiographic measure of chronic cardiac adaptation and a functional measure of anaerobic power in elite athletes. While direct comparisons are therefore limited, our findings align with the broader literature on concurrent training, which consistently shows that the greatest interference occurs in tasks requiring explosive power rather than maximal strength or endurance (Gomes et al. 2025; Huiberts et al. 2024; Manuel Clemente et al. 2021; Petre et al. 2021; Schumann et al. 2022). In a meta-analysis of 43 studies, concurrent training did not significantly reduce gains in maximal strength or muscle hypertrophy compared to strength training alone but resulted in smaller gains in explosive strength (Schumann et al. 2022). Another meta-analysis of 27 studies showed that compared to resistance training alone, concurrent training blunted gains in lower-body one-repetition maximum (1RM) for leg press and squat exercises among trained individuals (ES = − 0.35, *p* < 0.01). This finding indicates a significant interference effect, whereby endurance exercise compromises maximal strength adaptations in this specific population (Petre et al. 2021).

Recent meta-analytic evidence confirms that concurrent training differentially impacts adaptations in maximal strength versus explosive power. The interference effect on maximal lower-body strength development is characterized as small and appears to be specific to males. In contrast, the attenuation of explosive power is more consistent and pronounced, with a small-to-moderate interference effect observed in both males and females. This pattern, where power adaptations are more broadly and significantly blunted than maximal strength adaptations, supports the conclusion that explosive strength capabilities are more susceptible to compromise during a concurrent training paradigm (Huiberts et al. 2024). This pattern is further substantiated by recent meta-analytic evidence on the effects of high-intensity interval training (HIIT) in elite soccer players. A 2021 systematic review (Manuel Clemente et al. 2021) found that while various HIIT protocols consistently produced significant gains in endurance-related qualities, such as maximal oxygen uptake and repeated-sprint ability, the same programs failed to elicit significant improvements in key measures of explosive strength, including vertical jump height and linear sprinting time. This divergence in adaptation, where a training modality successfully enhances endurance capacity while concurrently showing a lack of efficacy for improving explosive power, provides a clear functional example of the interference effect (Manuel Clemente et al. 2021). This conclusion is reinforced by a recent randomized controlled trial that directly compared resistance-only (R) training with concurrent training in trained individuals. The study found that concurrent training significantly impaired adaptations in both maximal strength (measured by 1-repetition maximum) and explosive strength (measured by countermovement jump height) relative to the resistance-only group. This interference was particularly evident as the training program progressed, with maximal strength gains being curtailed in the concurrent training group during the later training phase.

The results of our study provide preliminary, experimental support that concurrent training creates a significant interference effect that potential compromises the development of distinct strength qualities (Gomes et al. 2025). The magnitude of the performance decrement observed is also practically significant. The present Δh_MAX_ (17%) between the high- and normal-LVMI groups corresponds to a 0.06 m decrement, comparable to the 0.04 difference that separates academy players who might do (e.g., professionals) and do not (e.g., U19) secure professional contracts (Papadakis et al. 2022a, b, c).

### Strengths and limitations

This study possesses several strengths that enhance the validity of its findings. Conceptually, it addresses a novel research question at the intersection of cardiovascular and neuromuscular physiology. Methodologically, the recruitment of a homogenous cohort of high-level professional soccer players (i.e., Greek Super League) from a single team minimizes confounding from variables such as coaching philosophy and tactical periodization, a critical factor for enhancing internal validity in applied sports science research (Manzi et al. 2023; Paolini et al. 2025). Finally, from an analytical standpoint, the application of a robust, multi-faceted statistical framework that triangulates findings across frequentist and Bayesian analyses bolsters confidence in the primary finding and aligns with modern calls for more rigorous, probabilistic statistical practices in the field (Gutierrez et al. 2025; Hammerton and Munafo 2021; Hecksteden et al. 2022; Solberg et al. 2025; Yung et al. 2025).

The findings of this study should be interpreted in light of several limitations. First, the homogeneous, all-male cohort from a single elite league enhances internal validity but limits generalizability. Whether a similar cardio-neuromuscular association exists in female athletes, who exhibit different patterns of cardiac remodeling (i.e., scaling and geometry in athletic LV mass), or across other competitive levels remains uncertain and requires dedicated investigation (Oxborough et al. 2024; Pelliccia et al. 2023; Unnithan et al. 2024). Second, the cross-sectional design establishes an association but precludes any inference of causality (Savitz and Wellenius 2023); it is plausible that the observed relationship is not a direct consequence of cardiac mass influencing power, but rather a reflection of underlying factors like training history or genetic predisposition (Lavin et al. 2022; Pelliccia et al. 2023). Third, the modest sample size (*n* = 19) is typical in elite cohorts, but confers low power for medium effects (35%), increasing Type II error risk and potentially obscuring smaller, yet meaningful relationships in secondary outcomes (Book et al. 2024; Murphy et al. 2025a, b; Warneke et al. 2025). Fourth, our analysis was limited to cardiac mass and did not include functional measures like global longitudinal strain (GLS), which can reveal subtle changes in myocardial function not captured by LVMI alone (Albaeni et al. 2021; Guo et al. 2025; Li et al. 2021; Wang et al. 2024). Similarly, the lack of direct physiological data, such as muscle fiber typology, prevents a deeper mechanistic explanation, as the proportion of fast-twitch fibers is a key determinant of explosive power (Egan and Sharples 2023; Methenitis et al. 2025; Roberts et al. 2023). Finally, while practical, M-mode echocardiography is known to be less accurate than 3D techniques for quantifying LV mass, particularly in athletes with non-standard ventricular geometries (Dissabandara et al. 2024; Kristensen et al. 2022). Lastly, although two experienced cardiologists analyzed the images, formal inter- and intra-observer reproducibility estimates were not calculated, which is a methodological limitation (Warneke et al. 2025).

### Practical applications and future directions

#### Practical applications

The basis of this investigation was grounded on the aforementioned research question of whether all physiological adaptations in elite athletes are independent. Findings highlight a cardio–neuromuscular link with implications for monitoring and programming**.** Where force plates are unavailable, photoelectric cells, contact mats, or validated smartphone apps can provide practical estimates of h_JUMP_ for longitudinal tracking (Montalvo et al. 2021; Stratford et al. 2020). Despite the fact that the above-mentioned methods are low cost compared to force plate recordings, they deprive the opportunity to measure key parameters such as V_GRF_ which can provide valuable information relating performance level and loading. The RVJT can thus serve as a functional, indirect proxy for the systemic balance of training adaptations where routine echocardiography is not feasible (Akyildiz et al. 2022; Bishop et al. 2023, 2022; Munoz-Gracia et al. 2025). For practitioners, a persistent, unexplained decline in h_MAX_, especially with stable RSI/P_REL_, should prompt consideration of high-velocity concentric micro-doses (e.g., loaded jump squats, plyometric complexes) to protect peak explosiveness (Bishop et al. 2023; Deely et al. 2022; Marques-Jimenez et al. 2022). Because this relationship is likely dynamic, a pragmatic cadence is advisable: pre-season baseline**,** mid-season check, and post-season assessment (Hostrup and Bangsbo 2023).

This information can be used to titrate training prescriptions more effectively. For example, to mitigate the interference effect, practitioners could implement periodized micro-cycles that strategically juxtapose hypertrophy-inducing endurance work with dedicated concentric power micro-doses, such as loaded jump squats or plyometric complexes (Feuerbacher et al. 2025; Hung et al. 2025; Ross et al. 2024; Stone et al. 2024; Wang and Bo 2024). Furthermore, to disentangle true concurrent training changes in neuromuscular function from changes in body composition, coaches should consider monitoring power output scaled to lean body mass rather than total body mass, providing a more precise assessment of an athlete's power-generating capacity (Ishida et al. 2021; Nobari et al. 2021; Ross et al. 2024; Stone et al. 2024; Theos et al. 2022; Wang and Bo 2024).

#### Future directions

Longitudinal and intervention trials should test whether changes in LVMI track with changes in h_MAX_, using adequately powered samples (≥ 55 participants based on our a priori estimate) and sex-inclusive cohorts. Incorporating advanced cardiac imaging (e.g., cardiac MRI/strain) and reporting inter-/intra-observer ICC will align with contemporary echo standards (Baba Ali et al. 2024; Bakogiannis et al. 2023; Grech and Abela 2025). Concurrently, incorporating direct measures of muscle architecture and molecular signaling would provide a clearer mechanistic picture of the proposed trade-off. Ultimately, this line of inquiry would benefit from adopting a Network Physiology approach, allowing for more sophisticated modeling of the dynamic interplay between the cardiovascular, neuromuscular, and metabolic systems. Such work would move the field toward a more integrated understanding of the elite athlete as a complex adaptive system with pleiotropic benefits (Balagué et al. 2022, 2024; Balague et al. 2022) that are reported through a variety of different disciplines and approaches (Garcia-Retortillo and Ch Ivanov 2024; Papadakis et al. 2022a, b, c).

## Conclusions

In conclusion, this study provides preliminary, cross-sectional evidence of an inverse relationship between left ventricular mass and explosive jump performance in elite male soccer players. Athletes with greater cardiac hypertrophy, a necessary adaptation to the sport's demands, exhibited a specific deficit in maximal jump height. This cardio-neuromuscular performance paradox underscores a potential trade-off between endurance and power adaptations, challenging practitioners to consider the integrated, consequences of their training prescriptions to optimize the delicate balance required for peak athletic performance.

## Supplementary Information

Below is the link to the electronic supplementary material.

Supplementary file1 (PNG 148 KB)

## Data Availability

The dataset generated and analyzed for this study can be found in the Open Science Framework (OSF) at the following URL: https://osf.io/ryjsp/.
