# Supplementary figures and images for: Stronger hearts, weaker leaps? The cardiac power paradox in elite soccer
Source: Eur J Appl Physiol. 2025 Oct 18;126(3):1463–78. doi: 10.1007/s00421-025-06026-3 (PMC13013311; doi:10.1007/s00421-025-06026-3)

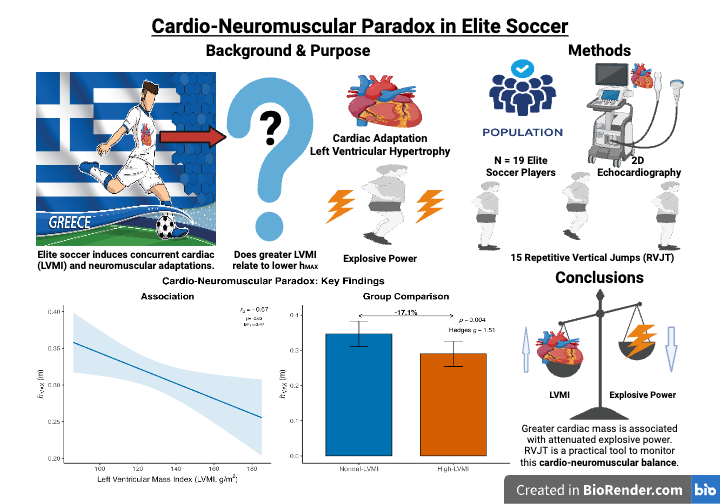

Supplement: Supplementary file 1 — Supplementary file1 (PNG 148 KB) [file 421_2025_6026_MOESM1_ESM.png]
